# Comparing the predictive discrimination of machine learning models for ordinal outcomes: A case study of dehydration prediction in patients with acute diarrhea

**DOI:** 10.1371/journal.pdig.0000820

**Published:** 2025-05-06

**Authors:** Kexin Qu, Monique Gainey, Samika S. Kanekar, Sabiha Nasrim, Eric J. Nelson, Stephanie C. Garbern, Mahmuda Monjory, Nur H. Alam, Adam C. Levine, Christopher H. Schmid

**Affiliations:** 1 Department of Biostatistics, Brown University, Providence, Rhode Island, United States of America; 2 Department of Emergency Medicine, Rhode Island Hospital, Providence, Rhode Island, United States of America; 3 Warren Alpert Medical School, Brown University, Providence, Rhode Island, United States of America; 4 International Centre for Diarrhoeal Disease Research Bangladesh, Dhaka, Bangladesh; 5 Emerging Pathogens Institute, University of Florida, Gainesville, Florida, United States of America; University of Ulm, Institute of Biomedical Engineering, Albert-Einstein-Allee 45, GERMANY, Ulm, 89081

## Abstract

Many comparisons of statistical regression and machine learning algorithms to build clinical predictive models use inadequate methods to build regression models and do not have proper independent test sets on which to externally validate the models. Proper comparisons for models of ordinal categorical outcomes do not exist. We set out to compare model discrimination for four regression and machine learning methods in a case study predicting the ordinal outcome of severe, some, or no dehydration among patients with acute diarrhea presenting to a large medical center in Bangladesh using data from the NIRUDAK study derivation and validation cohorts. Proportional Odds Logistic Regression (POLR), penalized ordinal regression (RIDGE), classification trees (CART), and random forest (RF) models were built to predict dehydration severity and compared using three ordinal discrimination indices: ordinal c-index (ORC), generalized c-index (GC), and average dichotomous c-index (ADC). Performance was evaluated on models developed on the training data, on the same models applied to an external test set and through internal validation with three bootstrap algorithms to correct for overoptimism. RF had superior discrimination on the original training data set, but its performance was more similar to the other three methods after internal validation using the bootstrap. Performance for all models was lower on the prospective test dataset, with particularly large reduction for RF and RIDGE. POLR had the best performance in the test dataset and was also most efficient, with the smallest final model size. Clinical prediction models for ordinal outcomes, just like those for binary and continuous outcomes, need to be prospectively validated on external test sets if possible because internal validation may give a too optimistic picture of model performance. Regression methods can perform as well as more automated machine learning methods if constructed with attention to potential nonlinear associations. Because regression models are often more interpretable clinically, their use should be encouraged.

## Introduction

Clinical prediction models can aid clinical decision-making [1–3]. The proliferation of electronic health records and genomic, proteomic, imaging and biomarker data has greatly increased the number of potential predictors and combinations of features that can be used to build models. When the number of candidate predictors becomes large, building models using traditional variable selection methods, such as stepwise regression, becomes challenging. It is not only easy to overfit models so that they do not perform well when tested on new data, but it is also difficult to fit flexible models that incorporate local effects, such as nonlinearity and interaction. Machine learning (ML) methods, such as random forest (RF) that average across individual trees constructed from different subsets of the predictors, or Least Absolute Shrinkage and Selection Operator (LASSO) and ridge regression that penalize large models, have been touted as ways to overcome the overfitting problem. These methods are now being increasingly used to construct clinical prediction models to support clinical decision-making [4].

On the other hand, regression models often are easier to interpret than machine learning methods because the predictions are based on a formula of known components easily converted into a simple score or integrated into a mobile health application. ML methods tend to have a black box character with hidden components. A systematic review found that models derived from LR were more transparent in reporting model performance than newer machine learning counterparts [5].

Recent studies comparing the performance of different machine learning models have reached conflicting conclusions. Some studies reported that machine learning approaches were worse than or no better than regression [6–9]; others found machine learning methods had better performance [10–17]. To date, most studies comparing the relative performance of machine learning and conventional regression models have focused on binary and continuous outcomes. However, outcomes with ordered categorical levels (e.g., good, fair, poor) are also common in medicine and healthcare. For instance, World Health Organization (WHO) guidelines recommend treating patients with acute diarrhea, one of the leading causes of death and disability in adults and children worldwide, based on three ordinal dehydration categories, with intravenous fluids given for severe dehydration, oral rehydration solution for some dehydration and expectant management only for no dehydration [18, 19]. Thus, it is also important to evaluate the performance of clinical prediction models for ordinal outcomes.

Many papers making these comparisons seem to use inadequate techniques for developing the regression models, however. Quite often, the regression method is not even described. For example, Mohammed *et al*. [20] compared logistic regression with gradient boosting, random forest and artificial neural networks to develop predictive models for three binary outcomes among patients undergoing total knee arthroplasty. They demonstrated that for each outcome the three machine learning methods performed about the same, but much better than logistic regression when examining different performance metrics. They selected features for their logistic regression models with backward selection (presumably stepwise regression, although this was not stated). Unstated, but implied, was that this selection only examined the main effects of the candidate predictors and ignored nonlinear effects or interactions. In another example, Pua *et al*. [21] developed an ordinal regression model predicting walking limitations following total knee arthroplasty that incorporated nonlinearity by modeling continuous outcomes as restricted cubic splines, but they did not include interactions in their models. In a systematic review, Christodoulou *et al*. identified 63 of 71 studies as not explicitly stating whether interaction effects were considered; the remaining 8 studies did not clear state their approach to interactions [7]. Because the power of machine learning methods arises from their ability to search for transformations and combinations of variables that best explain the outcomes, failing to allow regression models to see such transformations and combinations unfairly penalizes them. When regressions are developed in such a smart way, they can do just as well, if not better, than machine learning [22].

Another deficiency that arises in many comparisons of modeling techniques is a failure to externally validate model performance on data different from the one on which the model was trained. It is well-known that model performance declines when a model is transported to another setting because a model often reflects the local features of the data on which it was trained. A standard approach randomly splits the data into two parts. The model is developed on one part (training set) and validated on the second part (test set). Another approach uses cross-validation in which the data are divided into K parts, each of which serves as a test set for a model developed on the other parts. Overall model performance is then computed by averaging across the K test sets. This method of internal validation does account for some of the over-optimism of training model performance, but does not provide true external validation using an independently collected data set. External validation is widely accepted as providing a more accurate estimate of future model performance in practice and generalization to other settings, but is difficult to carry out because independent datasets are often unavailable [22–26].

Two systematic reviews of predictive models have reported that only a small subset of predictive models have ever been externally validated [7, 27]. One review noted that predictive performance using the external data was substantially reduced relative to that on training or internal validation [27]. The other review found that ML algorithms outperformed LR in studies with a high risk of bias, but not in studies with a low risk of bias. Risk of bias was defined as unclear or improper validation procedures; inconsistent application of data-driven variable selection; inconsistent handling of continuous predictors; use of different predictors for different methods; and differential application of corrections for imbalanced outcomes [7].

Thus, to date, it is unclear whether ML is superior to regression when regression models are developed appropriately, when proper external validation is available, and when outcomes are ordinal categories. Many of the studies cited above use inferior methods for logistic regression and do not have independently collected external validation sets.

The purpose of this manuscript is to compare the discrimination of models developed with ordinal LR (unpenalized and penalized), classification and regression tree (CART), and RF algorithms to predict ordinal dehydration severity (none, some, severe) in a single study of individuals presenting with acute diarrhea to a hospital in Dhaka, Bangladesh where independent training and test datasets were collected. Because interpretability is particularly important in medicine, we are interested in the relative performance of ordinal LR compared to other methods. Model performance is evaluated in (1) the original training dataset; (2) after adjusting for over-optimism using bootstrap resampling to approximate performance in an independent test set; and (3) after actual prospective validation in an independent test set [28–31].

## Materials and methods

### Data sources

Models were developed using data from the “Novel, Innovative Research for Understanding Dehydration in Adults and Kids” (NIRUDAK) Study. NIRUDAK was a prospective cohort study of adults and children over five years of age presenting with acute diarrhea (<7 days duration) to the rehydration unit at the International Centre for Diarrhoeal Disease Research, Bangladesh’s (icddr,b) Dhaka Hospital. Patients whose data were used for developing models were enrolled between March 2019 and March 2020 and patients involved in the validation study were enrolled from January to December, 2022. Table 1 summarizes characteristics of these two studies. The two datasets along with their metadata are freely available on the Open Science Framework at https://osf.io/pncms/.

**Table 1:** Details of the NIRUDAK derivation and validation studies. Both were prospective cohort studies recruiting adults and children 5 years and older at the the International Centre for Diarrhoeal Disease Research, Bangladesh’s (icddr,b) Dhaka Hospital.

Attribute	Derivation study	Validation study
Enrollment period	Mar 2019–Mar 2020	Jan 2022–Dec 2022
Study size	2172 patients	1601 patients
Participant selection	Random, stratified by age	Random
Missing outcome data	33 (1.6%) patients (26 missing outcome and 7 missing a predictor)	21 (1.3%) patients (all missing outcomes)
Complete data	2139 (98.4%) patients	1580 (98.7%) patients

Study staff randomly selected patients for screening for both studies on arrival to Dhaka Hospital 24 hours per day, 7 days per week. If the patients randomly selected for screening met study eligibility criteria, research staff provided the patient or their parent or guardian with information about the goals, risks, and benefits of the study and obtained written consent in Bangla. For children aged 11–17 years, verbal or written assent was also obtained in addition to consent from their patient or guardian. Patients who could not read provided verbal consent and a thumb stamp on the consent form in lieu of their signature. Verbal or written consent was obtained for all enrolled patients.

All patients in both studies were assessed on arrival for 14 clinical signs and symptoms of dehydration that were chosen a priori based on the available literature [32]. After measurement of initial weight and completion of clinical assessment, all patients were managed according to standard icddr,b protocols and weighed every four hours until discharge. Demographic and social data were obtained from either the patient or guardian.

Percent weight change with rehydration was used as the criterion standard for dehydration, based on standards in the literature [33–35]. Percent dehydration was calculated as 100×[(Stable Weight−Admission Weight)/Stable Weight]. Stable weight was calculated by averaging the two highest consecutive weights that differed by less than 2%. Patients who did not achieve a stable weight prior to discharge were asked to return for a final post-illness weight measurement when their diarrhea resolved. This final weight was used instead of the stable weight in the formula above. Patients were categorized as having severe (>9%), some (3–9%), or no (<3%) dehydration, as recommended in the literature [36–38].

### Statistical methods

This study developed models predicting the ordinal outcome of dehydration severity using four predictive techniques: proportional odds logistic regression (POLR), random forest (RF), RIDGE (penalized) regression, and classification and regression trees (CART). We chose these particular methods as each of them had packages available in R that could fit ordinal regression models using the variable selection algorithms we employed. In particular, we could not fit LASSO models because the ordinal regression package we used, *ordinalNet*, could not retain all levels of categorical predictors and lower-order terms of variables involved in interactions. Creating new code that would allow for such hierarchical variable selection is needed, but is beyond the scope of this project. We also searched through Python libraries and found no packages that could solve this issue either.

Ten-fold cross validation on the training data optimizing method-specific criteria detailed in the supplementary material S1 Text for each method was adopted to determine the tuning parameters for each method to avoid over-fitting (Algorithm 1): model size (number of main effects and interactions) for forward stepwise variable selection with POLR, shrinkage parameter λ for RIDGE, model complexity parameters *cp* and *minsplit* for CART, and number of variables randomly sampled as candidates at each split *mtry* for RF. The optimized tuning parameters were then used to form the final models. All methods were implemented using available packages in R 3.6.3 as described below.

**Algorithm 1. Algorithm to train models using K-fold cross-validation**

 1. Divide data randomly into *K* equally sized sets $S_{1},\ldots ,S_{K}$

 2. Define a reasonable grid {$\alpha_{1},\alpha_{2},\ldots ,\alpha_{J}$} of *J* values for the tuning parameter α.

 3. For k=1,…,K

 (a) Use *S*_*k*_ as holdout test data and other *K*–1 sets as training data.

 (b) For j=1,…,J:

  i. Construct model for $\alpha_{j}$ on training data and evaluate model performance using log-likelihood *E*_*kj*_ with model-specific criteria, on *S*_*k*_.

 4. Compute average test performance ${\overset{―}{E}}_{j}$ for j=1,…,J across *K* holdout test sets as ${\overset{―}{E}}_{j}=\frac{1}{K}\sum_{k=1}^{K}E_{kj}$ and choose $\alpha_{m}$ where $m={\text{argmax}}_{j}{\overset{―}{E}}_{j}$ as optimal value for α.

 5. Construct the final model on the whole data set with $\alpha =\alpha_{m}$.

Candidate predictors for the models included 8 categorical clinical predictors and 6 continuous predictors (Table 2). Before modeling, 3 continuous variables were converted to categorical scales to avoid problems related to clustering of values at round numbers: number of vomiting episodes (none, 1–4, 5–9, >10); number of diarrheal episodes (3–9, 10–19, >19); and duration of diarrhea (0–12, 13–23, <23 hours) [39]. The thresholds were chosen based on both the observed data distribution and clinical relevance. Categorical variables were modeled using a set of indicator variables relative to a chosen normal reference level. The following four sets of variables were considered for POLR and RIDGE: (1) categorical variables and linear forms of continuous variables; (2) set one plus all two-way interactions of variables in set one; (3) categorical variables and restricted cubic splines using three knots at the $10^{\text{th}}$, $50^{\text{th}}$, $90^{\text{th}}$ quantiles for continuous variables; (4) set three and all two-way interactions of variables in set three. CART and RF models considered only set one given the automated way that interactions and nonlinear relationships are captured in these models.

**Table 2:** Candidate predictors

Categorical predictors	
**Variable**	**Levels**
Mental status	Normal, Confused/Lethargic, Unconscious
Thirst	Normal, Drinks eagerly, Refuses/Unable to Drink
Skin pinch	Rapid, Slow, Very slow
Eyes	Normal, Sunken
Respirations	Normal, Deep
Radial pulse	Strong, Decreased, Absent
Capillary refill	Normal, Prolonged
Urine output	Normal, Decreased/Dark, Minimal/None
Sex	Male, Female
# Vomiting episodes	<1, 1 – 5, 6 – 10, >10
# Diarrheal episodes	<10, 10 – 19, >19
Duration of diarrhea (hours)	<13, 13 –23, >23
**Continuous predictors**	
Age	
Supine systolic blood pressure	
Mid-upper arm circumference	


#### Proportional odds logistic regression (POLR).

We developed two algorithms described in section 1 of the supplementary material S1 Text for fitting forward stepwise ordinal proportional odds logistic regression models with a cumulative logit link with the *polr* function in the MASS package in R [40]. Algorithm A in S1 Text developed models without interactions and Algorithm B developed models with interactions. Both algorithms used the log-likelihood as the performance measure to choose the tuning parameter for selecting the number of variables to include in the model. Models with interaction terms were developed by examining all the pairwise interactions of each set of main effects chosen in the forward steps and used a similar strategy to choose a tuning parameter for the number of interactions to retain. We also fit non-proportional odds logistic regression models, but as the results were similar to POLR (Brant-wald test for non-proportionality p = 0.1), we chose to report only the POLR model results.

#### Ridge regression (RIDGE).

Ridge regression is a regularization technique that introduces a small amount of bias in order to reduce model variance and the chances of overfitting. Regression coefficients are modified to optimize a penalized likelihood parameterized by a tuning (penalty) parameter λ [41]. We fit a cumulative logit ridge regression for the ordinal outcome using the *ordinalNetTune* function in the *ordinalNet* R package [42]. In order to compare with the proportional odds form of the regression, we assumed parallel-form models (i.e., identical non-intercept coefficients across all outcome categories). Section 2 in supplementary material S1 Text provides technical details.

#### Recursive partitioning (CART).

Classification trees for ordinal responses were implemented using the *rpartScore* function in the *rpartScore* R package [43]. *RpartScore* implements recursive partitioning by fully growing a tree and then pruning it. The program uses two parameters to control the size of the tree and minimize overfitting. *Minsplit* controls tree growth by setting the minimum number of observations a node must contain before it can be split. Small values of *minsplit* produce larger trees. *Cp* is a cost-complexity parameter that penalizes the tree for its size by weighting tree size with goodness of fit. Both parameters were tuned with 10-fold cross validation. After pruning, each observation was assigned a predicted category corresponding to the category most frequently occurring among all the observations in the terminal node to which the observation was assigned. See section 3 and Algorithm C in S1 Text for technical details.

#### Random forest (RF).

We implemented the *ordfor* function in the *ordinalForest* R package that fits random forests to ordinal responses [44]. We tuned the number of variables available for splitting at each node (*mtry*), and chose the optimal value as that giving the maximum average log-likelihood across the 10 cross-validation folds. For comparability with other methods that return a predicted probability, we used the predicted probability score as the performance measure within *ordfor*. To avoid overfitting that may arise due to tuning only the *mtry* parameter, a sensitivity analysis was conducted by individually tuning each of the remaining parameters (*nsets*, *ntreeperdiv*, *ntreefinal*, *npermtrial*, *bnest*, and *min.node.size*) while keeping the others at their default values. As we did not find the algorithm to be sensitive to changing these parameters, we left them at their default values. Section 4 in S1 Text provides technical details.

#### Model assessment.

We assessed the ability of each model to discriminate between K ordinal categories using three ordinal discrimination indices that generalize the binary c-statistic to ordered polytomous outcomes: generalized c-index (GC), ordinal c-index (ORC) and average dichotomous c-index (ADC) [45]. As with binary classifications, a value of 0.5 is equivalent to a model that discriminates no better than random chance, while a value of 1 represents a model with perfect discrimination. Section 5 in S1 Text provides details.

To correct for overoptimism of discrimination measured on the training data, we applied Harrell’s method [28], Efron’s 0.632+ bootstrap [29] and Efron’s original 0.632 method [30]. Each algorithm compensates for overestimation on the training set by weighting discrimination on the original training data with discrimination on an out-of-bag bootstrap test set. Section 6 and Algorithm D in S1 Text provide details.

Each model was externally validated on the test dataset using all three c-indices. 95% confidence intervals were calculated using bootstrap.

### Ethical statement

Ethical approval for both these studies was obtained from Rhode Island Hospital Institutional Review Board (1244580; 1764819) and the icddr,b Ethical Review Committee (PR-18077; PR-21048).

## Results

The NIRUDAK training dataset included 2172 patients with acute diarrhea, of whom 2139 (98.4%) had complete data, including 431 (20.1%) with no dehydration, 1431 (66.9%) with some dehydration, and 277 (12.9%) with severe dehydration. The NIRUDAK validation dataset included 1601 patients with acute diarrhea, of whom 1580 (98.7%) had complete data, including 301 (19.1%) with no dehydration, 1159 (73.4%) with some dehydration, and 120 (7.6%) with severe dehydration. Because the amount of missing data was small, we used only the complete cases for analysis. Table 3 provides a summary of the patient sociodemographic and clinical data collected in both training and testing datasets. Several differences are apparent. Patients in the training dataset were older, more male, less highly educated, had lower incomes and were more severely dehydrated than those in the test dataset.

**Table 3:** Baseline sociodemographic and clinical data

	Training data (*N* = 2139)	Test data (*N* = 1580)
Age (years)^a^	35.0 (18.0–60.0)	28.0 (21.0–39.0)
Female sex^b^	1063 (49.7)	921 (58.3)
Urban (vs. rural/suburban)	628 (76.1)	1159 (73.4)
Years of education	3.0 (0.0–7.0)	5.0 (2.0–9.0)
Monthly household income (USD)	168.0 (120.0–240.0)	180.0 (120.0–40.0)
Nutritional status		
Severe acute malnutrition	31 (1.4)	10 (0.6)
Moderate acute malnutrition	164 (7.7)	160 (10.1)
No acute malnutrition	1944 (90.9)	1410 (89.2)
Dehydration		
Severe	277 (12.9)	120 (7.6)
Some	1431 (66.9)	1159 (73.4)
None	431 (20.1)	301 (19.1)

^a^Median (25th-75th percentiles) for continuous variables

^b^Number (Percent) for categorical variables

Separate models were fit using POLR, RF, RIDGE, and CART (Table 4). For POLR and RIDGE, we show only the model form with the best performance (models including interactions but without splines in both cases). On the full training dataset, RF had the best performance, followed by POLR, RIDGE, and CART. The final POLR model, however, included only 10 variables, as compared to the RF, RIDGE, and CART models, which included all 14 variables. Performance for each ordinal model was slightly higher when measured using ORC and slightly lower when measured using GC, but the overall trend was similar.

**Table 4:** Performance of four modeling methods using three discrimination indices, including average dichotomous c-index (ADC), ordinal c-index (ORC), and generalized c-index (GC), applied to the original training dataset, three different bootstrap methods of internal validation, and to the testing dataset. 95% confidence intervals computed from 1000 bootstrap samples.

Index	Model	Train	Harrell	Efron 0.632	Efron 0.632+	Test
ADC	POLR	0.79	0.77(0.75,0.79)	0.77(0.75,0.78)	0.76(0.74,0.78)	0.71(0.68,0.74)
	RF	0.92	0.86(0.85,0.87)	0.81(0.79,0.82)	0.78(0.76,0.80)	0.65(0.62,0.68)
	RIDGE	0.79	0.75(0.73,0.76)	0.76(0.75,0.78)	0.76(0.74,0.78)	0.60(0.58,0.63)
	CART	0.77	0.68(0.65,0.74)	0.72(0.69,0.75)	0.71(0.68,0.75)	0.67(0.64,0.70)
ORC	POLR	0.81	0.77(0.75,0.79)	0.78(0.76,0.80)	0.78(0.76,0.80)	0.74(0.71,0.77)
	RF	0.90	0.87(0.86,0.89)	0.83(0.81,0.85)	0.81(0.79,0.83)	0.66(0.63,0.68)
	RIDGE	0.81	0.75(0.74,0.77)	0.78(0.76,0.79)	0.77(0.76,0.79)	0.62(0.59,0.65)
	CART	0.78	0.66(0.63,0.69)	0.70(0.67,0.73)	0.68(0.65,0.72)	0.72(0.69,0.75)
GC	POLR	0.77	0.75(0.73,0.78)	0.75(0.73,0.77)	0.75(0.73,0.77)	0.69(0.66,0.72)
	RF	0.87	0.78(0.77,0.79)	0.78(0.76,0.80)	0.77(0.75,0.78)	0.63(0.60,0.66)
	RIDGE	0.77	0.73(0.72,0.75)	0.75(0.73,0.76)	0.74(0.73,0.76)	0.60(0.57,0.63)
	CART	0.75	0.65(0.61,0.71)	0.69(0.67,0.72)	0.69(0.65,0.72)	0.67(0.64,0.70)

When the four models were adjusted for overoptimism using Efron’s 0.632+ bootstrap method, performance decreased substantially for RF, somewhat less for CART, and even less for POLR and RIDGE. While the adjusted performance of POLR and RIDGE were similar to the performance in the full training dataset, the performance of CART and RF were lower because of their poor performance on the bootstrap out-of-bag test data. The relative ordering of the model results was similar regardless of the adjustment method used to correct for overoptimism although the differences between them varied.

Performance for all four models was lower using the test data compared with adjusted and unadjusted performance on the training data, though performance decreased more for RIDGE and RF than POLR and CART. On the final test data, the POLR model performed best, followed by CART, RF, and RIDGE.

The sensitivity analysis showed that the RF algorithm was robust to hyperparameter tuning, achieving the same model performance on training and test datasets regardless of which hyperparameters were tuned through cross-validation.

## Discussion

Although internally and externally validated clinical predictive models and studies comparing the relative performance of machine learning and conventional regression methods for clinical predictive models have become more common in the past decade, few have examined ordinal outcomes, which are also important in clinical medicine. Even fewer, if any, have constructed the regression model with proper consideration of nonlinear effects and statistical interactions and have then compared performance on an external, independent data set.

We have compared the discrimination of four machine learning models for predicting the ordinal outcome of no, some, or severe dehydration in patients over five years presenting with acute diarrhea to a hospital in Bangladesh. All models considered the same variables and all two-way interactions. Performance was measured in the derivation set [32], with internal validation using bootstrapping, and in an external validation set collected in the same clinical setting but several years later [46].

On the original training dataset from which the four models were developed, RF had better discrimination than the other methods. POLR, RIDGE and CART performed similarly to each other, although POLR required the fewest predictors, an important factor for busy clinicians using the model to guide clinical management. After adjustment for over-optimism (over-fitting) using various bootstrap techniques, performance declined significantly for RF and CART, while decreasing only slightly for POLR and RIDGE. Model performance on an external test set declined substantially for all models, although most severely for RF and RIDGE. Performance on the test set was best for POLR, with CART next. The substantial decline of RF and RIDGE may result from the over-complexity of these models, which involve all predictors and two-way interactions, despite being tuned during training to prevent overfitting. As a sensitivity analysis, we trained a new RIDGE model on the external test set and found that the tuning parameter and estimated coefficients for RIDGE changed significantly.

While in most cases AUC area for each of the three discrimination indices was highest when models were applied to the training set and lowest when applied to the external test set, performance varied considerably across the different internal validation methods except for POLR for which the Harrell, Efron and Efron 0.632 and Efron 0.632+ methods gave similar results for each discrimination index. RIDGE results also varied little across the three internal validation methods, although performance was highest with Efron 0.632 and lowest with Harrell’s method. However, estimated performance of RF and CART varied considerably by method. The Harrell method gave much higher estimates for RF with the ADC and ORC indices, but not for the GC index; conversely, for CART Harrell’s method gave lower estimates for all three indices. The two Efron methods varied much less. For ORC, all of the internal validation methods overadjusted for CART relative to performance on the test set.

The unusual results for CART arise because CART actually performs much better on the bootstrap samples than on the training sample. CART is therefore overadjusted in Harrell’s method. This seems to occur because bootstrap samples contain a large number of duplicates (about 36.8% on average). When a tree is built, duplicate observations end up in the same leaf of the final tree, leading to overfitting of these duplicates. The Efron methods only use out-of-bag predictions from the bootstrap samples and thus do not suffer from this overfitting.

It is also important to note that the POLR models required the fewest predictors which facilitates their use in clinical practice. Indeed, an important factor not often discussed regarding clinical prediction models is their ease of use by clinicians, who must take time to assess or gather each of the predictors necessary for the model. The more predictors that must be gathered, the less likely a clinician might be to use the model in practice, even if its discrimination is very good. Similarly, clinicians may also be more willing to use more intuitive methods such as POLR and CART, which allow them to understand how each of the predictors contributes to the model’s estimate of the outcome, rather than more black box methods such as RF.

Proponents of ML methods often tout their ability to learn about variable interactions algorithmically and point to this feature as an important reason for their superiority over older regression-based approaches. But since the nature of these interactions is not readily apparent in the model output, many users may not feel comfortable with a model whose form is not completely transparent to them. Our results here demonstrate that a regression algorithm that incorporates intelligent searching for variable interactions can give results that are not only more interpretable, but also superior to those from ML algorithms, at least in problems where the number of candidate predictors is small relative to the number of samples. In problems with more predictors, the search for interactions may become less computationally feasible, but then one might try a hybrid approach in which the ML algorithms are used to identify candidate interactions which can then be fed into the regression variable selection algorithm [47].

Evaluating performance with a dataset used to develop a clinically important predictive model provides an important empirical comparative test of these models unavailable from simulated data. The generalizability of our conclusions are, however, limited by the use of one dataset with particular features. In particular, the sample size of NIRUDAK was relatively large and the number of candidate predictors relatively small. While many clinical studies are of this type because they tend to include candidate predictors that have been prescreened as likely to be clinically relevant, many studies do have many more predictors than individuals. Machine learning approaches may well perform better in those scenarios. Second, external validity ideally requires the training and test datasets to come from different settings. Both sets in this example were collected at the same hospital in Dhaka, Bangladesh. This limitation is mitigated somewhat by their being collected several years apart and differing substantially in terms of several important sociodemographic variables, such as age, sex, education, income, and the overall percentage of patients with some and severe dehydration.

We focused on model discrimination because several methods have been described in the literature and packages were available in R to assess and compare discrimination for ordinal models. Discrimination is also likely most important for prediction studies such as this one where clinicians must rapidly assign patients to a diagnostic category based on information at the bedside [45]. Other common measures for assessing performance of prediction models, such as calibration, decision curves and net benefit are beyond the scope of this paper as software is not currently available for calculating these measures for ordinal models, but are worth examining in subsequent studies [26]. Moreover, given that discrimination on an external validation set is poor for the ML models, one would also expect calibration to be poor. In any case, one would not address calibration until better discrimination could be achieved. In addition, we focused this study on the subset of machine learning methods for which software packages are currently available for handling both ordinal models and ordinal predictors. Future efforts should be made to develop similar software packages for ordinal models using other machine learning methods, such as LASSO, support vector machines, and neural networks, so that they can be compared to ordinal logistic regression models as well.

## Conclusion

Despite its excellent discriminatory performance on the training data, RF and RIDGE performance degraded after adjustment for over-optimism and validation in a test dataset, while POLR largely maintained its original good performance. CART performed as well as POLR on the training dataset, but similar to RF its performance decreased after adjustment for over-optimism and external validation. POLR may have the added benefits of being more efficient (with fewer predictors for the clinician to assess), more likely to validate well, and more easily integrated into common clinical decision support tools such as mobile health applications and paper-based clinical scores. Given the inability of current methods to fully adjust for overoptimism, it remains important for all clinical prediction models to be externally validated in a test dataset prior to being recommended for clinical use.
